# Unraveling RUNX2 mutation in a cleidocranial dysplasia patient: Molecular insights into osteogenesis and proteostasis

**DOI:** 10.1016/j.gendis.2024.101449

**Published:** 2024-11-06

**Authors:** Luca Dalle Carbonare, Arianna Minoia, Alberto Gandini, Francesca Cristiana Piritore, Cristina Patuzzo, Lucrezia Ceretti, Anna Vareschi, Antonino Aparo, Mattia Cominacini, Giovanni Malerba, Maria Grazia Romanelli, Joao Pessoa, Daniele Guardavaccaro, Franco Antoniazzi, Maria Teresa Valenti

**Affiliations:** a Department of Engineering for the Innovation Medicine, University of Verona, Verona 37100, Italy; b Department of Surgery, Dentistry, Pediatrics and Gynecology, University of Verona, Verona 37100, Italy; c Department of Neurosciences, Biomedicine and Movement Sciences, University of Verona, Verona 37100, Italy; d Research Center LURM (Interdepartmental Laboratory of Medical Research), University of Verona, Verona 37100, Italy; e Department of Medical Sciences and Institute of Biomedicine-iBiMED, University of Aveiro, Aveiro 3810-193, Portugal; f Department of Biotechnology, University of Verona, Verona 37134, Italy

Runt-related transcription factor 2 (RUNX2), also called core-binding factor subunit alpha-1 (CBFA1), is the bone-specific transcription factor considered the master gene in osteogenesis, contains a crucial RUNT domain for DNA binding, and is regulated by multiple mechanisms. During the initial stages of osteogenesis, the expression levels of RUNX2 are primarily elevated and then gradually decrease during the formation of osteoblasts and osteocytes.^1^ Abnormal levels of RUNX2 can severely affect osteoblasts and skeletal structure. *RUNX2* mutations are linked to cleidocranial dysplasia (CCD), a rare autosomal dominant skeletal disorder characterized by abnormal skeletal phenotypes.^2^ Our previous data demonstrate that *RUNX2* mutations alter the modulation of p53 levels.^2^ However, despite the documented involvement of the RUNX2 protein in numerous cellular pathways beyond osteogenic differentiation, there are no in-depth studies on the impact of these mutations on cellular homeostasis. To explore the effects of mutations in the RUNT domain of RUNX2, here, we examined the cellular impact of the *RUNX2* mutation c.505C > T in induced mesenchymal stem cells (iMSCs) obtained from induced pluripotent stem cells derived from one 26-year-old female CCD patient (Fig. S1). Our research provides the first molecular studies of this *RUNX2* mutation in a CCD patient.

The CCD patient enrolled in the present study exhibited short stature, flat feet, and mild lumbar spine and hip demineralization, but with no scoliosis or vertebral fractures. X-ray and dual-energy X-ray absorptiometry scans showed decreased bone mineral densities and T scores (Fig. S2A–E). The patient also had baby teeth and vitamin D deficiency. Peripheral blood analysis of the CCD patient revealed a c.505C > T mutation in the *RUNX2* gene, causing an R169W missense mutation in the RUNT domain of the RUNX2 protein, which is essential for osteogenesis (Fig. S2F–H).

Initially, we analyzed the expression of mRNAs related to osteogenesis in circulating progenitor cells from this *RUNX2*-mutated CCD patient and compared it with two sex- and age-matched healthy controls. The array analysis revealed several differentially expressed mRNAs in the osteogenic pathway (Table 1S). Notably, transforming growth factor beta 1 (TGF-β1) expression was significantly higher in the *RUNX2*-mutated patient than in the healthy donors, along with up-regulation of insulin-like growth factor 1 receptor (IGF1R) and TGF-β receptor 1 (TGF-βR1) (Fig. 1A). The gene expression dendrogram showed TGF-β1 forming a distinct cluster, separate from other genes (Fig. S3A). Quantitative reverse-transcription PCR confirmed increased mRNA levels of TGF-β1 and TGF-βR1 in the *RUNX2*-mutated patient, compared with healthy controls (Fig. S3B). After generating *RUNX2*-mutated iMSCs, assessing their morphology and pluripotency (Fig. 1B–E), and confirming the overexpression of TGF-β1 and TGF-βR1 in these cells (Fig. 1F), we further investigated the gene regulation mechanisms potentially associated with TGF-β1 and TGF-βR1 up-regulation. miR-9-5p, which regulates TGF-β1, TGF-βR1, and RUNX2,^3^ was decreased in *RUNX2*-mutated circulating progenitor cells and iMSCs (Fig. 1G). Silencing miR-9-5p in mesenchymal stem cells decreased both mRNA and protein levels of RUNX2 and increased TGF-β1 and TGF-βR1 expression (Fig. S4A, B). To evaluate the effects of the *RUNX2* mutation on osteogenic differentiation, we cultured both control and *RUNX2*-mutated iMSCs under osteogenic stimuli. After three days, *RUNX2* mRNA levels continued to increase in wild-type iMSCs but stabilized in *RUNX2*-mutated cells (Fig. 1H). RUNX2 protein levels decreased in wild-type cells from the third day to the seventh day while remaining stable in mutated cells, indicating impaired osteogenic differentiation in the latter condition (Fig. 1I).

**Figure 1:**
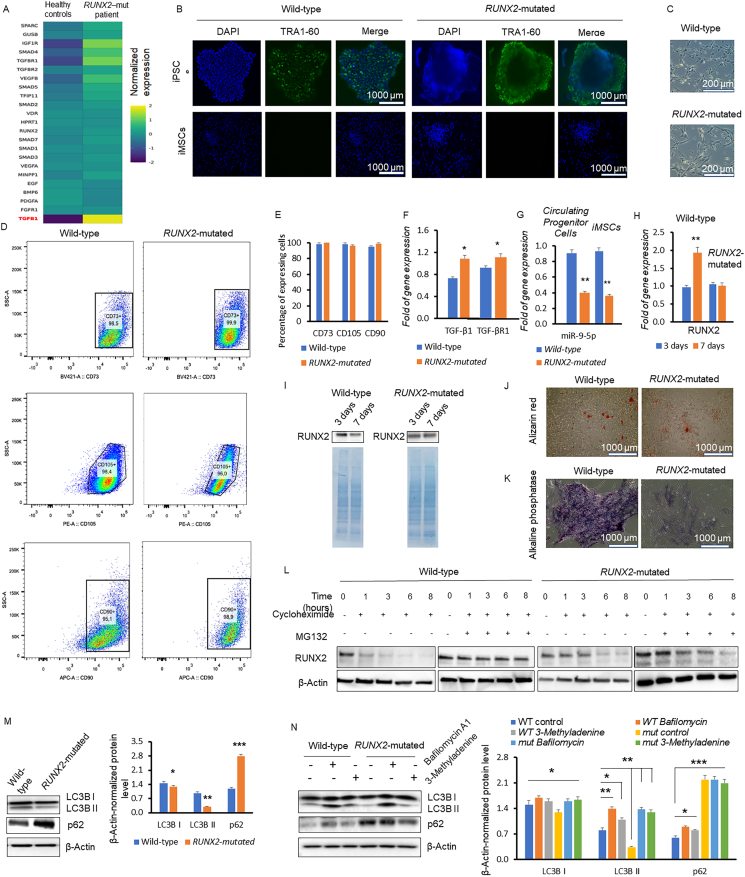
Altered gene expression, proteasome degradation, and autophagy in Runt-related transcription factor 2 (RUNX2)-mutated cells associated with cleidocranial dysplasia (CCD). **(A)** The heatmap illustrates gene expression differences between the CCD patient and the healthy donors. Gene expression values were normalized by subtracting the mean expression value of each gene (calculated as the average between its expression in healthy donors and the patient), followed by z-score normalization. **(B)** Fluorescent TRA1-60 staining of wild-type and *RUNX2*-mutated induced pluripotent stem cells (iPSCs) and induced mesenchymal stem cells (iMSCs). The scale bar represents 1000 μm. **(C)** Fibroblast-like morphology of wild-type and *RUNX2*-mutated iMSCs visualized by phase-contrast microscopy. The scale bar represents 200 μm. **(D)** Flow cytometry analyses of the stem cell markers CD105, CD73, and CD90 in iMSCs. In each analysis, the fluorescence intensity of the labeled cellular marker (*x*-axis) and the side scatter area (*y*-axis) were measured. The percentages of iMSCs expressing each protein are indicated in the respective gated subpopulation. **(E)** Percentages of wild-type and *RUNX2*-mutated iMSCs expressing CD105, CD73, and CD90 obtained by FlowJo software. **(F)** Quantitative reverse-transcription PCR (RT‒qPCR) quantifications of transforming growth factor beta 1 (TGF-β1) and transforming growth factor beta receptor 1 (TGF-βR1) in wild-type and *RUNX2*-mutated iMSCs. **(G)** RT‒qPCR analysis of miR-9-5p expression in wild-type and *RUNX2*-mutated circulating progenitor cells and iMSCs. The results were normalized against U6 snRNA. **(H, I)** RT‒qPCR analysis of mRNA levels (H) and western blotting analysis of the protein levels (I) of RUNX2 during osteogenic differentiation of wild-type and *RUNX2*-mutated iMSCs after three and seven days of differentiation stimulation. In panel I, SDS‒PAGE of total protein extracts is shown under each Western blot. **(J, K)** Images of wild-type and *RUNX2*-mutated iMSCs stained with alizarin red (the calcification loci were stained in red) (the scale bar represents 1000 μm) and alkaline phosphatase activity (the levels of which were decreased in *RUNX2*-mutated iMSCs) (the scale bar represents 1000 μm). **(L)** Western blotting visualization of RUNX2 protein levels in wild-type and *RUNX2*-mutated iMSCs treated with cycloheximide (alone or in combination with the MG132 proteasome inhibitor) for 1 h to 8 h. (−: without cycloheximide or MG132; +: in the presence of cycloheximide or MG132). **(M)** Western blots (left) and densitometry analyses (right) of the protein levels of the microtubule-associated proteins 1A/1B light chain 3B (LC3B) and p62/sequestosome 1 (SQSTM1) in wild-type and *RUNX2*-mutated iMSCs. **(N)** Western blots (left) and densitometry analyses (right) of LC3B and p62/SQSTM1 protein levels after three days of osteogenic differentiation, in wild-type and *RUNX2*-mutated iMSCs, untreated (controls) or treated with bafilomycin A1 or 3-methyladenine. ∗*P* < 0.05, ∗∗*P* < 0.005, ∗∗∗*P* < 0.001.

Observing the increase in the expression of downstream genes of RUNX2 from the third day to the seventh day, we found that wild-type iMSCs exhibited higher expression levels of RUNX2 downstream genes than mutated cells (Fig. S5). Thus, an important implication of the lack of modulation of *RUNX2* is a reduction in maturation level, as evidenced by the decreased levels of calcification nodules indicated by alizarin red staining and alkaline phosphatase expression (Fig. 1J, K). After characterizing the impact of the *RUNX2* mutation on osteogenic differentiation, we sought to elucidate the molecular mechanisms contributing to the sustained levels of RUNX2 protein in *RUNX2*-mutated cells during osteogenesis, from the third (early phase) to the seventh (middle phase) day of differentiation. After three days of osteogenic differentiation, the degradation of RUNX2 (evaluated by inhibiting protein synthesis with cycloheximide) was observed in wild-type iMSCs, where RUNX2 levels progressively decreased over time (Fig. 1L, left panel). When cycloheximide was combined with the MG132 proteasome inhibitor, RUNX2 levels remained stable over time (Fig. 1L, center-left panel). When the assay was repeated in cells carrying mutated RUNX2, treatment with cycloheximide resulted in decreased degradation of RUNX2 (Fig. 1L, center-right panel) relative to that of the wild-type protein (Fig. 1L, left panel). Furthermore, the combination of cycloheximide with MG132 resulted in some degradation of RUNX in *RUNX2*-mutated iMSCs (Fig. 1L, right panel), which was not observed for the wild-type protein under the same conditions (Fig. 1L, center-left panel). These observations indicate that wild-type RUNX2 was mostly degraded by the proteasome. Nevertheless, mutant RUNX2 not only showed a slightly increased cellular half-life but also seemed to be degraded by other cellular processes, since it was still degraded under proteasomal inhibition. Therefore, we focused on another system involved in protein degradation, autophagy. Autophagy and the ubiquitin–proteasome system are two primary degradation systems involved in cellular homeostasis. Both systems communicate with each other and function in conjunctions to maintain proteostasis and organelle homeostasis.^4^

The expression of autophagy-related gene 5 (ATG5) and autophagy-related gene 7 (ATG7), which are autophagy-promoting proteins, was lower in both iMSCs (Fig. S6A) and circulating progenitor cells (Fig. S6B) carrying the *RUNX2* mutation, relative to wild-type cells. Accordingly, the protein levels of the microtubule-associated proteins 1A/1B light chain 3B II (LC3B II) and p62 autophagy markers were decreased and increased, respectively, in *RUNX2*-mutated iMSCs, compared with wild-type cells (Fig. 1M), suggesting a reduction in autophagy in *RUNX2*-mutated iMSCs. These results indicate that the alternative proteasome-independent degradation pathway of mutant RUNX2 was likely not autophagy. They also suggest that the slightly increased half-life of mutant RUNX2 could result in its decreased autophagic degradation.

To investigate whether reduced autophagy could be due to a block in autophagic flux, we inhibited autophagy using bafilomycin and 3-methyladenine. As shown in Figure 1N, although LC3 II levels were lower in mutated iMSCs, autophagy blockade resulted in increased levels of this protein, indicating reduced autophagy. However, p62 levels remained unchanged under autophagy inhibition, contributing to autophagic impairment. In addition to reduced autophagy, this finding suggests proteasome failure. It has been demonstrated that accumulated p62 sequesters ubiquitinated proteins, thereby delaying their transportation toward the proteasome.^5^ On the other hand, STRING analysis indicated an association between p62/SQSTM1 (sequestosome 1) and TGF-β1, which was overexpressed in *RUNX2*-mutated cells (Fig. S7). Bioinformatic analysis revealed significant interactions among RUNX2, TGF-β1, TGF-βR1, MAP1LC3B (microtubule-associated protein 1 light chain 3 beta), and p62/SQSTM1 proteins (*P* = 0.00977). The functional enrichment analysis (protein–protein interaction enrichment) revealed several biological processes involved, including regulation of odontogenesis (GO:0042481), cartilage development (GO:0061035), cranial skeletal system development (GO:1904888), and skeletal system morphogenesis (GO:0048705). Importantly, functional protein interaction studies have revealed the following disease-related gene associations: osteoarthritis [DOID:8398], bone remodeling disease [DOID:0080005], and bone disease [DOID:0080001].

Limitations of our work include the study of *RUNX2* mutations in samples derived from a single CCD patient. Overall, our findings provide a comprehensive exploration of how the *RUNX2* c.505C > T mutation impacts molecular and cellular pathways, contributing insight into the pathogenesis of CCD and related skeletal disorders.

## CRediT authorship contribution statement

**Luca Dalle Carbonare:** Writing – review & editing, Investigation, Conceptualization. **Arianna Minoia:** Investigation. **Alberto Gandini:** Investigation. **Francesca Cristiana Piritore:** Investigation. **Cristina Patuzzo:** Investigation. **Lucrezia Ceretti:** Investigation. **Anna Vareschi:** Investigation. **Antonino Aparo:** Investigation. **Mattia Cominacini:** Investigation. **Giovanni Malerba:** Investigation. **Maria Grazia Romanelli:** Writing – review & editing. **Joao Pessoa:** Writing – review & editing. **Daniele Guardavaccaro:** Conceptualization. **Franco Antoniazzi:** Conceptualization. **Maria Teresa Valenti:** Writing – review & editing, Writing – original draft, Supervision, Investigation, Conceptualization.

## Ethics declaration

The study was conducted according to the guidelines of the Declaration of Helsinki and approved by the local ethical committee of Azienda Ospedaliera Integrata Di Verona (protocol code: 1538; 3 December 2012). Informed consent was obtained from all the subjects involved in the study.

## Conflict of interests

The authors declared no competing interests.

## Figures and Tables

**figs1:**
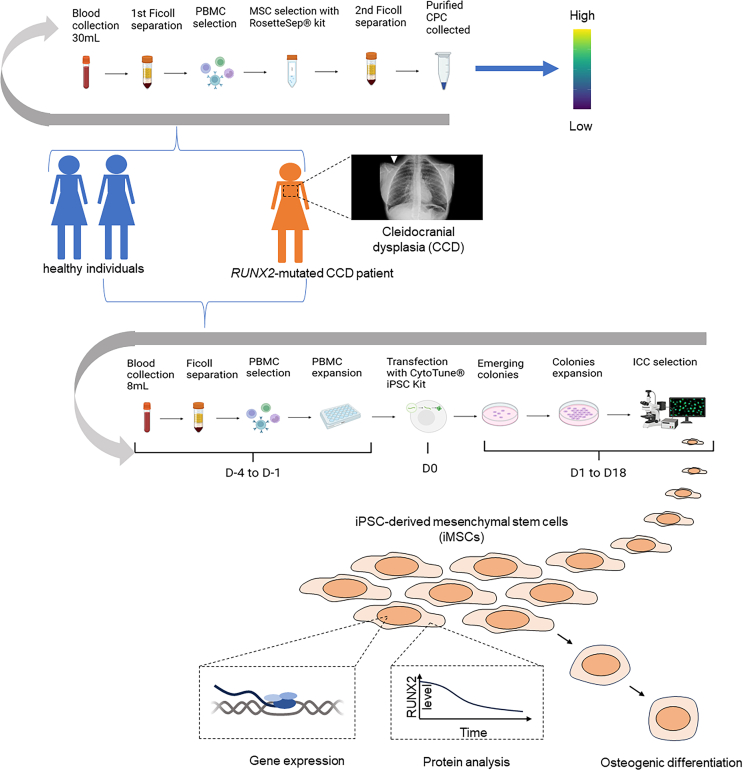


**figs2:**
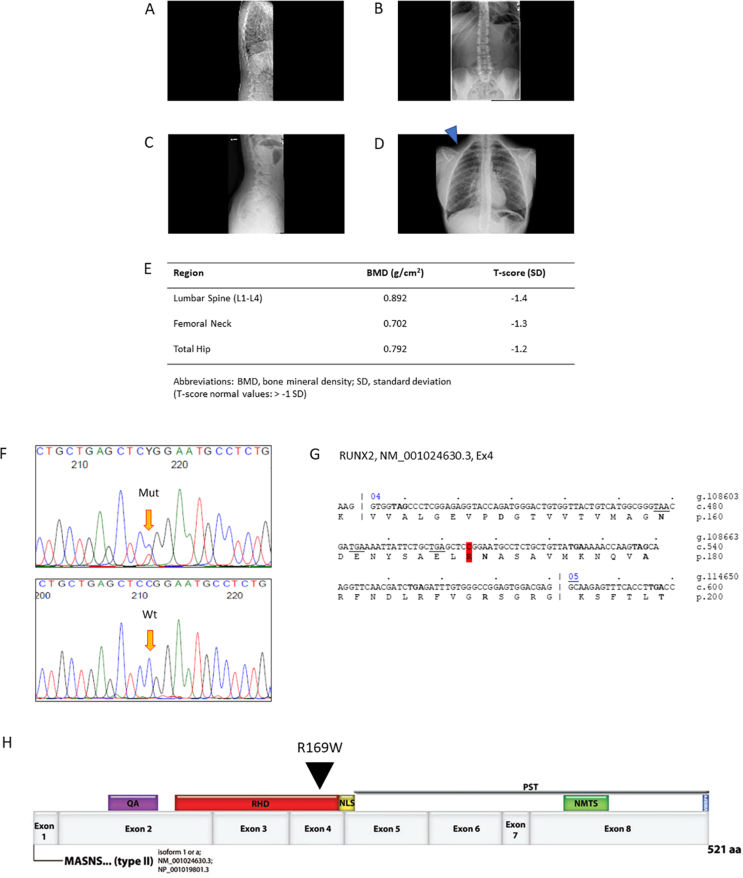


**figs3:**
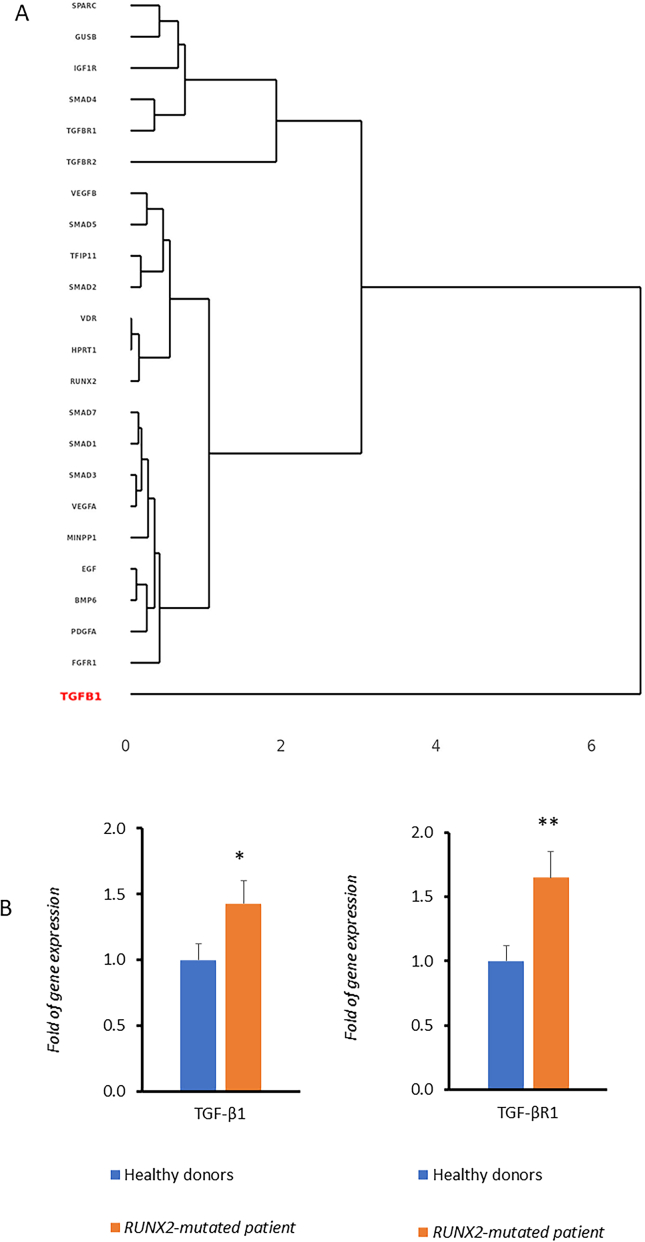


**figs4:**
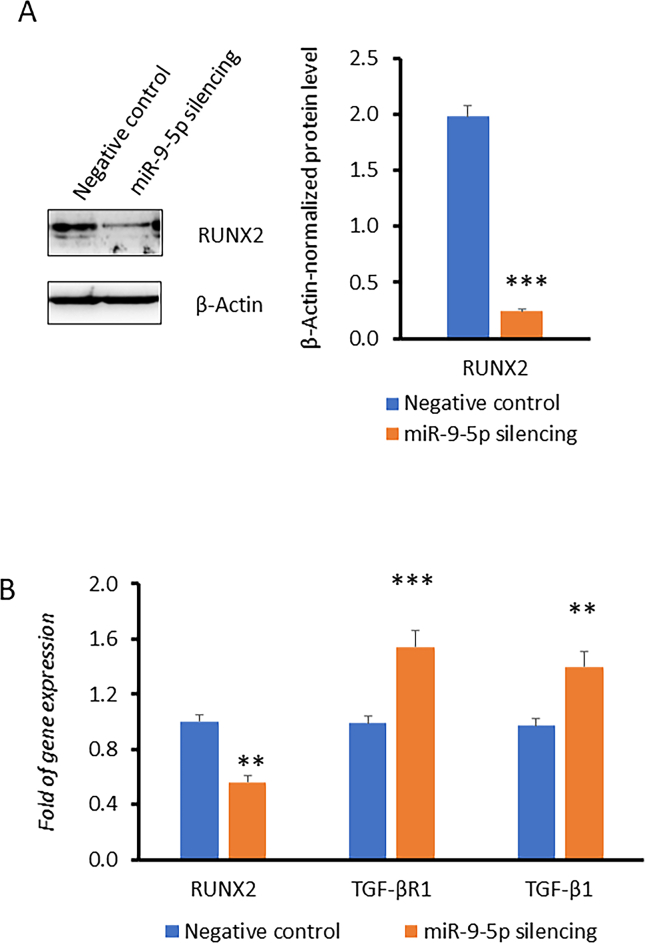


**figs5:**
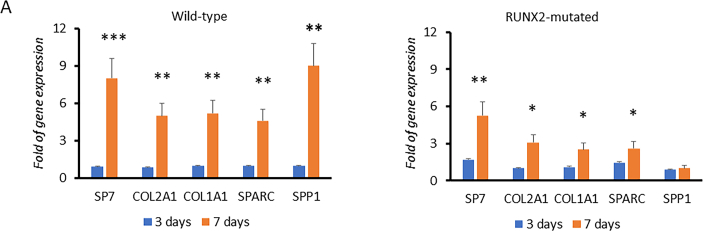


**figs6:**
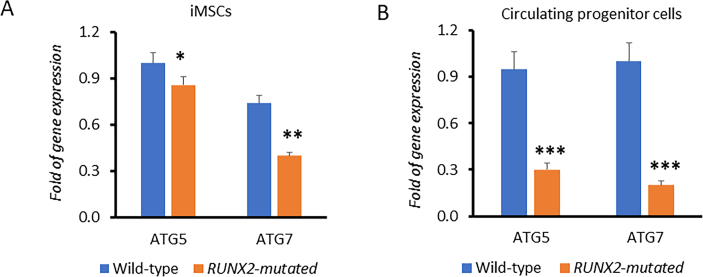


**figs7:**
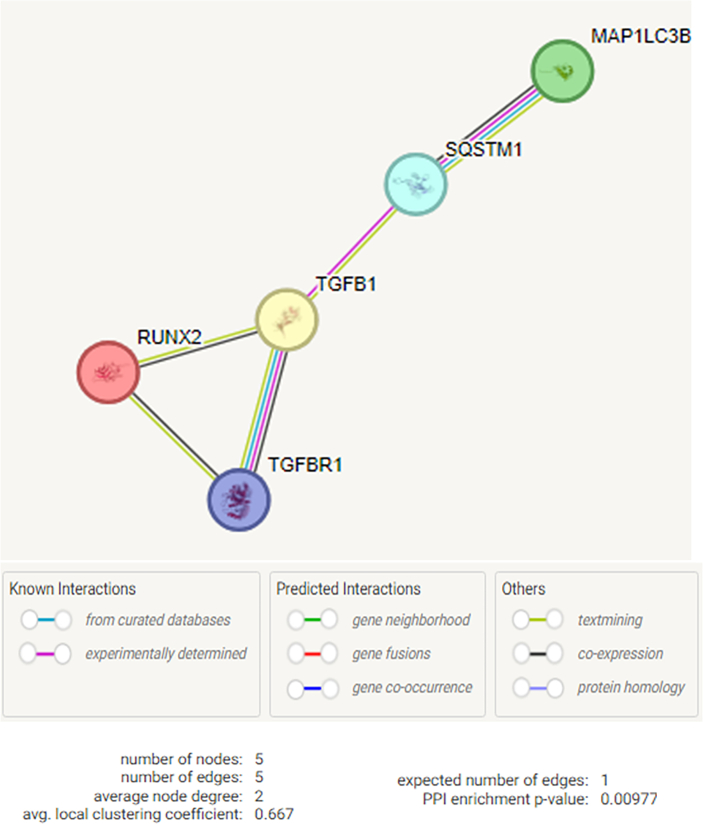


## Data Availability

All the data generated or analyzed during this study are included in this published article or the *Supplemental Materials*.
