# Supplementary material for: Unraveling RUNX2 mutation in a cleidocranial dysplasia patient: Molecular insights into osteogenesis and proteostasis
Source: Genes Dis. 2024 Nov 6;12(4):101449. doi: 10.1016/j.gendis.2024.101449 (PMC11960636; doi:10.1016/j.gendis.2024.101449)
Supplement: Multimedia component 1 [file mmc1.docx]

**Materials and methods**

**Experimental Workflow**

In the present study, blood samples were collected from one CCD patient carrying the *RUNX2* c.505C>T mutation and two age-matched healthy individuals to isolate circulating progenitors. Mesenchymal stem cells derived from induced pluripotent stem cells (iMSCs) were generated from the blood samples of a healthy donor and a CCD patient. IMSCs were used to study the impact of *RUNX2* mutation on osteogenic differentiation, as well as on RUNX2 protein levels and associated gene expression alterations. The workflow is visually summarized in **Figure 1**.

**Subjects**

Peripheral blood samples were collected from a patient carrying a *RUNX2* mutation (female, 32 years old) diagnosed with CCD and two healthy donors (females, 25 and 26 years old). Informed consent was obtained from each donor. The study was approved by the ethical committee of Azienda Ospedaliera Universitaria Integrata of Verona, Italy (number 1538; 3 December 2012; local ethical committee of Azienda Ospedaliera Integrata di Verona).

**DNA Extraction**

Cells were subjected to DNA extraction utilizing the QIAamp DNA Blood Mini Kit (Qiagen, Milan, Italy) according to the manufacturer's instructions. Subsequent DNA quantification was carried out using a Qubit 4 Fluorometer (Invitrogen, Thermo Fisher Scientific, Waltham, MA, USA) and a Qubit dsDNA HS Assay Kit (Invitrogen, Thermo Fisher Scientific, Waltham, MA, USA).

**Sanger Sequencing**

The *RUNX2* mutation was identified by targeted Sanger sequencing. A specific primer pair (purchased from Thermo Fisher Scientific, Waltham, MA, USA) with a relative amplicon spanning the entire length of *RUNX2* exon 4 was designed with Primer-BLAST (https://www.ncbi.nlm.nih.gov/tools/primer-blast/index.cgi? LINK_LOC=BlastHome; accessed on 03/02/2019). The sequences of primers used were as follows: forward, AGTGGCATCACAACCCATACA; reverse, AGAAAAACACTCAACTTCATCTGG. PCR was performed with the Mastercycler® ep Gradient S® (Eppendorf, Milan, Italy) and the GoTaq® Hot Start Polymerase kit (Promega, Madison, WI, USA) under the following conditions: 2 minutes at 96 °C; 35 cycles at 96 °C for 30 seconds, 60 °C for 30 seconds, and 72 °C for 30 seconds; and a final step at 72 °C for 5 minutes. The quality of the PCR products was assessed using 1.5% agarose gel electrophoresis. After purification using the FastGene™ kit (Nippon Genetics, Mariaweilerstraße, Düren, Germany), 1 µL of the purified PCR product was sequenced with Dye Terminator Cycle Sequencing (DTCS) using the Quick Start Kit and a CEQ8000 Genetic Analysis System (Sciex, Milan, Italy) following the manufacturer’s instructions.

**Circulating progenitor cells (CPCs)**

CPCs were obtained as we previously described (10). Briefly, blood samples were collected from each subject and mixed with phosphate-buffered saline (PBS; Lonza Inc., Walkersville, MD, USA) at a 1:1 ratio. The mixture was gently poured over a Ficoll-PaqueTM PLUS (GE, Healthcare Biosciences AB, Uppsala, Sweden) solution (at a 2:1 ratio) for cell stratification through density gradient centrifugation (800 g, 30 minutes, 20 °C). Peripheral blood mononuclear cell (PBMC) rings containing monocytes, lymphocytes, and mesenchymal stem cells (MSCs) were collected, washed with PBS, and treated with RosetteSepTM (RosetteSep Mesenchymal Enrichment Cocktail; cat. number: 15128, StemCells, Vancouver, BC, Canada) for 20 minutes.

The RosettaSep-treated mixture underwent a second Ficoll-Plaque-based centrifugation (at a 2:1 ratio, 800 × g, 30 minutes, 20 °C) to remove hematopoietic cells. Purified cells were collected, washed with PBS, and subjected to a final PBS-washing step with centrifugation (15 minutes, 21,000 g, 20 °C). The resulting pellet was analyzed or stored at -80 °C until use.

**RNA extraction and reverse transcription**

Total RNA was extracted using an RNeasy® Protect Mini Kit (Qiagen, [Hilden, Germany](https://www.google.com/search?client=firefox-b-d&sca_esv=7978873cf7ff4aec&sxsrf=ADLYWIKcJgf1XHrcYqtaYs1VgaY2jx1O0g:1715093278597&q=Hilden&stick=H4sIAAAAAAAAAONgVuLUz9U3MI43yU16xGjCLfDyxz1hKe1Ja05eY1Tl4grOyC93zSvJLKkUEudig7J4pbi5ELp4FrGyeWTmpKTmAQC5rf7VTQAAAA&sa=X&ved=2ahUKEwixkIKt5PuFAxU7ywIHHZPUDeUQzIcDKAB6BAgXEAE)) following the manufacturer's protocol. For microRNA extraction, the miRNeasy Qiagen Mini Kit (Qiagen, [Hilden, Germany](https://www.google.com/search?client=firefox-b-d&sca_esv=7978873cf7ff4aec&sxsrf=ADLYWIKcJgf1XHrcYqtaYs1VgaY2jx1O0g:1715093278597&q=Hilden&stick=H4sIAAAAAAAAAONgVuLUz9U3MI43yU16xGjCLfDyxz1hKe1Ja05eY1Tl4grOyC93zSvJLKkUEudig7J4pbi5ELp4FrGyeWTmpKTmAQC5rf7VTQAAAA&sa=X&ved=2ahUKEwixkIKt5PuFAxU7ywIHHZPUDeUQzIcDKAB6BAgXEAE)) was used. The quantity and quality of the RNA samples were assessed using the ‘Qubit™ RNA HS assay kit’ (Invitrogen, Thermo Fisher Scientific, Waltham, MA, USA) and a Qubit 3 Fluorometer (Invitrogen, Thermo Fisher Scientific, Waltham, MA, USA; REF Q3321). Total RNA reverse transcription was performed with a first-strand cDNA synthesis kit (GE, Healthcare Biosciences AB, Uppsala, Sweden) following the manufacturer's protocol. MicroRNAs were transcribed using the TaqMan MicroRNA Reverse Transcription Kit (Thermo Fisher Corporation; Waltham, MA, USA; cat. no. 4366596) following the manufacturer's protocol. The thermocycler steps were performed using a GeneExplorer^TM^ Thermal Cycler.

**Osteogenic gene expression array**

mRNA expression profiling was performed using the TaqMan™ array human osteogenesis kit (Thermo Fisher Corporation, Waltham, MA, USA) according to the manufacturer’s instructions. Four mRNA assay endogenous controls included in the array were used for data normalization, and the ∆∆Ct method was used to evaluate the fold change.

**Gene expression analysis by real‐time PCR**

Real-time PCR was performed using TaqMan Universal PCR Master Mix (Thermo Fisher Scientific, Waltham, MA, USA) with the following commercially designed primers and probes and iTAQ Universal SYBR Green SuperMix (Bio-Rad, Hercules, CA, USA):

**List of probes/primers used in the present study.**

| Target gene | Supplier | Code/sequence |
| --- | --- | --- |
| RUNX2 | Applied Biosystems | Hs1047973_m1 |
| β_2_-Microglobulin | Applied Biosystems | H200187842_m1 |
| β-Actin | Applied Biosystems | Hs99999903_m1 |
| SP7 | Applied Biosystems | Hs00541729_m1 |
| SPP1 | Applied Biosystems | hs00167093_m1 |
| SPARC | Applied Biosystems | Hs00234160_m1 |
| COL2A1 | Applied Biosystems | [Hs00264051_m1](https://www.thermofisher.com/taqman-gene-expression/product/Hs00264051_m1?CID=&ICID=&subtype=) |
| COL1A1 | Applied Biosystems | [Hs00164004_m1](https://www.thermofisher.com/taqman-gene-expression/product/Hs00164004_m1?CID=&ICID=&subtype=) |
| TGF-βR1 | Applied Biosystems | Hs00610320_m1 |
| miR-9-5p | Applied Biosystems | 000583 |
| U6 snRNA | Applied Biosystems | 001973 |
| ATG3 forward | Invitrogen – Thermo Fisher Scientific | 5’-TGGAAGTGGCTGAGTACCTG-3’ |
| ATG5 forward | Invitrogen – Thermo Fisher Scientific | 5’-CACAAGCAACTCTGGATGGGATTG-3’ |
| ATG5 reverse | Invitrogen – Thermo Fisher Scientific | 5’-GCAGCCACA GGACGAAACAG-3’ |
| ATG7 forward | Invitrogen – Thermo Fisher Scientific | 5’-AGATTGTCCTAAAGCAGTTG-3’ |
| ATG7 reverse | Invitrogen – Thermo Fisher Scientific | 5’-CCATACATTCACTGAGGTTC-3’ |
| β-Actin forward | Invitrogen – Thermo Fisher Scientific | 5’-GAAGGATTCCTATGTGGGCG-3’ |
| β-Actin reverse | Invitrogen – Thermo Fisher Scientific | 5’-GGTCTCAAACATGATCTGGGT-3’ |
| TGF-β1 forward | Invitrogen – Thermo Fisher Scientific | 5’-GGAAATTGAGGGCTTTCGCC-3’ |
| TGF-β1 reverse | Invitrogen – Thermo Fisher Scientific | 5’-CCGGTAGTGAACCCGTTGAT-3’ |

Both the housekeeping genes β-actin and β_2_-microglobulin were used to normalize mRNA gene expression, while U6 was used to normalize miRNA expression.

Next, 19 µL of PCR mixture was added to the wells of a MicroAmp Optical 96-well reaction plate (Applied Biosystems, Thermo Fisher Scientific, Waltham, MA, USA), followed by the addition of 1 µL of cDNA. Three replicates of each sample were loaded. Following a brief centrifugation, the plate was covered and sealed with an appropriate membrane (Opti-seal, AB Analitica, Padova, Italy). For this procedure, the Line Gene 9620 Real-Time PCR System (Aurogene, Hangzhou Bioer Technology, Hangzhou, China) was used. The PCR conditions included an initial denaturation step, followed by 40 amplification cycles. At least three independent analyses were performed, and the Ct values were averaged. The fluorescence signals (fluorescein amidite (FAM), 2’-chloro-7’-phenyl-1,4-dichloro-6-carboxy-fluorescein (VIC), or SYBR Green) were captured and analyzed using a real-time PCR system. Subsequently, the expression levels of the target genes were normalized to those of appropriate reference genes. Relative gene expression levels were subsequently determined using the comparative cycle threshold (Ct) method: relative RNA level = 2^−(ΔCt target − ΔCt reference)^.

**Bioinformatics**

To generate informative heatmaps that can distinguish genes exhibiting different expression levels, the final heatmap was designed to focus on genes that showed significant variability in expression between the *RUNX2*-mutated CCD patient and the healthy controls. This selection criterion was empirically established, setting a threshold of 0.2 for the difference in expression levels between the patient and the healthy controls, resulting in the identification of 19 genes within this category of high variability. The complete list with gene expression values is provided in the supplementary material (Supplemental Table 2).

By acknowledging the significant diversity in expression levels among genes, a preprocessing step was incorporated. Each gene expression value was adjusted by subtracting the mean expression value, which was computed as the average between the gene expression in the control and patient samples. This adjustment aimed to visually accentuate expression disparities for each gene across healthy and diseased conditions. Following this step, a z score normalization was applied to standardize the expression values.

Additionally, gene ordering across all heatmaps was achieved through hierarchical clustering, with accompanying dendrograms provided for each experimental folder. This clustering approach facilitated the grouping of genes based on their expression similarity, which was computed using the Euclidean distance with complete linkage clustering (12). The entire analysis was conducted using R.

**Induced pluripotent stem cells (iPSCs)**

We obtained induced pluripotent stem cells (iPSCs) from peripheral blood samples of healthy donors and CCD patients via venipuncture using the CytotuneTM – iPS 2.0 Sendai Reprogramming Kit according to the manufacturer’s instructions (Thermo Fisher Scientific, Waltham, MA, USA; catalog numbers: A16517, A16518). After PBMC extraction using the aforementioned procedure, these cells underwent a transduction process involving vectors based on a modified, nontransmissible form of Sendai virus (SeV).

The reprogramming factor genes introduced through this procedure were essential for inducing the cells to enter an artificial pluripotent stem cell-like state, closely resembling that of an embryonic stem cell. The experiment was executed by visualizing the cells using the EVOS M7000 Core Imaging System (Invitrogen, Thermo Fisher Scientific, Waltham, MA, USA), and the cells were subsequently incubated at 37 °C in a 5% CO_2_ humidified atmosphere.

**Immunofluorescence**

Immunofluorescence analysis was performed following the manufacturer’s protocol for the Stem Cell Antibody Kits for Live Cell Imaging (Thermo Fisher Corporation, Waltham, MA, USA; cat. number: A24879). Briefly, we centrifuged the dye‑conjugated antibody solution TRA1-60 (Thermo Fisher Corporation, Waltham, MA, USA; cat. number: A25618). We added a 1:50 volume of the dye‑conjugated antibody directly to the cell culture medium of the cells to be stained and mixed by gentle swirling, incubated for 30 minutes at 37 °C and then removed the staining solution and gently washed the cells 2–3 times with FluoroBrite™ (Cat. Number: A1896701, Thermo Fisher Corporation, Waltham, MA, USA). For optimal results, we took images immediately (within 30 minutes) using the EVOS XL Core Imaging System (Invitrogen, Thermo Fisher Scientific-Thermo Fisher Corporation, Waltham, MA, USA).

**iPSC-derived mesenchymal stem cells (iMSCs)**

The iPSC-derived mesenchymal stem cells (induced MSCs, iMSCs) were derived from iPSCs, as previously reported (13). The cells were cultured in MesenPro RSTM Basal Medium (Gibco-Thermo Fisher,  [Thermo Fisher Corporation, Waltham, MA, USA](https://www.google.com/search?client=firefox-b-d&sca_esv=d0537759349b5ac4&sxsrf=ACQVn0-3oZoEwdP8Nei48g-sNb1-jUOuiA:1706993288457&q=Waltham&stick=H4sIAAAAAAAAAONgVuLQz9U3yKgyNn3EaMwt8PLHPWEprUlrTl5jVOHiCs7IL3fNK8ksqRQS42KDsnikuLjgmngWsbKHJ-aUZCTmAgDLoZJyTAAAAA&sa=X&ved=2ahUKEwi9od7EhZCEAxWMhf0HHcBEDuMQzIcDKAB6BAgZEAE); cat. number 12746012) supplemented with 2% MesenPro RSTM Growth Supplement (Gibco-Thermo Fisher, Thermo Fisher Corporation, Waltham, MA, USA; cat. number 12746012), 1% PSA antibiotics (penicillin, streptomycin, amphotericin B solution; Biological Industries Sartorius, [Gottingen, Germany](https://www.google.com/search?client=firefox-b-d&sca_esv=d0537759349b5ac4&sxsrf=ACQVn0_lNCbvAzFbYb1lIs82Z5Z2YD2juA:1706993439329&q=Gottinga&stick=H4sIAAAAAAAAAONgVuLQz9U3SMoyMXjEaMwt8PLHPWEprUlrTl5jVOHiCs7IL3fNK8ksqRQS42KDsnikuLjgmngWsXK455eUZOalJwIAuPdnUk0AAAA&sa=X&ved=2ahUKEwiH7NaMhpCEAxU8VfEDHYd_DcMQzIcDKAB6BAgVEAE)), and 1% L-glutamine (Gibco-Thermo Fisher, Thermo Fisher Corporation, Waltham, MA, USA; cat. number A2916801). The cells were incubated at 37 °C in a 5% CO_2_ humidified atmosphere.

**Cytofluorimetric analyses**

Cytofluorometric analyses were conducted using a BD LSRFortessa™ X-20 Cell Analyzer (BD Biosciences, Becton, Dickinson and Company Corporation, Franklin Lakes, NJ, USA), which enables the differentiation of distinct cellular surface molecules for immunophenotyping. Following cell expansion in flasks, accutase (Biowest, Bradenton, FL, USA; cat number: L0950-100) was used for harvesting after centrifugation. The pellet was resuspended in PBS and subjected to cell counting with Trypan Blue (Gibco-Thermo Fisher Corporation, Waltham, MA, USA; cat. number: 15250061) staining using a Countess II cell analyzer (Thermo Fisher Corporation, Waltham, MA, USA). Approximately 200,000 cells were used for each cluster differentiation (CD) marker analysis. After determining the live cell counts, a buffer solution was added, and specific antibodies (CD73-Bv421; BD Biosciences, Becton, Dickinson and Company Corporate, Franklin Lakes, NJ, USA; cat. number: 562430), CD90-APC (BD Biosciences, Becton, Dickinson and Company Corporate, Franklin Lakes, NJ, USA; cat. number: 561971), and CD105-PE (BD Biosciences, Becton, Dickinson and Company Corporate, Franklin Lakes, NJ, USA; cat. number: 560839) were added to the tubes and incubated for 40 minutes at 4 °C. After centrifugation, the pellet was resuspended and analyzed using an instrument. Viability was assessed through propidium-conjugated phycoerythrin (PE) (eBioscience Thermo Fisher, Thermo Fisher Corporation, Waltham, MA, USA; cat. number: BMS500PI) staining and autofluorescence quantification (for the negative control). FlowJo software (BD Biosciences, Becton, Dickinson and Company Corporation, Franklin Lakes, NJ, USA) was utilized for the cytofluorimetric imaging data analysis.

**Cell transfection**

Human mesenchymal stem cells (hMSCs, PromoCell, Heidelberg, Germany) were plated into a culture plate flask and cultured in MesenPRO RS Basal Medium for MSCs containing a 10% supplement mix (Gibco, Life Technologies Corporation, Grand Island, NE, USA; cat. number: 12747-010) and 1% penicillin/streptomycin/amphotericin.

When the cell confluence reached 60%-70%, transfection was carried out using Lipofectamine 3000 reagent (Invitrogen by Thermo Fisher Scientific Baltics UAB, Vilnius, Lithuania; cat. number: L3000‐008). To downregulate miR-9-5p (hsa-miR-9-5p, Thermo Fisher Corporation, Waltham, MA, USA; cat. number: [4427975](https://www.thermofisher.com/order/catalog/product/4427975)) expression, cells were transfected with a miR-9-5p inhibitor (mirVana, miRNA inhibitor- Ambion by Life Technologies, Thermo Fisher Corporation, Waltham, MA, USA Cat. Number 4464084, ID:MH10022) along with a scramble-negative control (Ambion by Thermo Fisher Scientific, Thermo Fisher Corporation, Waltham, MA, USA; cat. number: AM17010) according to the manufacturer’s instructions. At 24 hours posttransfection, the cells were collected, and RNA or protein extraction was performed as previously reported (14).

**Osteogenic differentiation**

The cells were cultured under a humidified atmosphere of 5% CO_2_ in StemPro® Medium (Gibco™, Thermo Fisher Corporation, Waltham, MA, USA) supplemented with osteogenesis (Gibco, Life Technologies Corporation, Grand Island, NE, USA; cat. number: A10069-01) and 1% penicillin, streptomycin, and amphotericin B solution (Biological Industries, [Gottingen, Germany](https://www.google.com/search?client=firefox-b-d&sca_esv=d0537759349b5ac4&sxsrf=ACQVn0_lNCbvAzFbYb1lIs82Z5Z2YD2juA:1706993439329&q=Gottinga&stick=H4sIAAAAAAAAAONgVuLQz9U3SMoyMXjEaMwt8PLHPWEprUlrTl5jVOHiCs7IL3fNK8ksqRQS42KDsnikuLjgmngWsXK455eUZOalJwIAuPdnUk0AAAA&sa=X&ved=2ahUKEwiH7NaMhpCEAxU8VfEDHYd_DcMQzIcDKAB6BAgVEAE)). After differentiation, the cells were harvested using accutase, washed, and used for cellular and molecular analyses. To assess the autophagy flux, cells were exposed to 10 nM bafilomycin A1 (Sigma-Aldrich, St. Louis, MO, USA) or 1 mM 3-methyladenine (Sigma, Shanghai, China) for 6 hours, following established protocols.

**Alizarin Red S staining**

Alizarin red staining (Sigma‒Aldrich, St. Louis, MO, USA; cat. number: A5533-25G) was performed following a 14-day culture period under osteogenic stimulation, as previously reported (15). Briefly, cells were fixed with 70% ethanol at room temperature. After washing, the cells were stained with 40 mM Alizarin red S for 5 minutes at pH 4.1, rinsed for 15 minutes with 1x PBS buffer, and evaluated under an optical microscope.

**Alkaline Phosphatase Staining**

Alkaline phosphatase (ALP) staining was conducted after a 21-day culture period under osteogenic stimulation. Briefly, cells were fixed with 4% paraformaldehyde in PBS (Gibco, Thermo Fisher Corporation, Waltham, MA, USA) for 2 minutes. Following fixation, the cells were washed with 1x PBS (Gibco, Thermo Fisher Corporation, Waltham, MA, USA). Subsequently, the cells were incubated for 15 minutes at room temperature in the dark with BCIP/NBT Color Development Substrate (Promega Corporation, Madison, WI, USA; cat. number: S3771) prepared according to the manufacturer’s instructions. Following the incubation period, the cells were once again washed with 1× PBS (Gibco, Thermo Fisher Corporation, Waltham, MA, USA) and then examined for staining under an optical microscope.

**Western blotting**

The protein concentration was determined using the BCA Protein Assay Kit (Quantum Protein, Bicinchoninic Protein Assay, **Euroclone S.p.A.** Milan, Italy) and measured with the VICTOR Microplate Reader (PerkinElmer, Waltham, MA, USA). Subsequently, protein samples were normalized with Pierce™ 1X radioimmunoprecipitation buffer (Thermo Fisher Corporation, Waltham, MA, USA; cat. number: 78501) and loading buffer. After boiling at 99 °C for 7 minutes, the samples were loaded onto precast Mini-PROTEAN® TGX gels (Bio-Rad, Hercules, CA, USA) for SDS‒PAGE, which utilized two gels for Coomassie Brilliant Blue (Sigma‒Aldrich, St. Louis, MO, USA; cat. number: 1154440025) staining and electroblotting. SDS‒PAGE was conducted in 1X running buffer for 1 hour. Proteins were transferred to a polyvinylidene fluoride membrane (Invitrogen, Thermo Fisher Corporation, Waltham, MA, USA; cat. number: 88518), activated with ethyl alcohol and water, and immersed in 1X transfer buffer for 15 minutes. Transfer was performed using a Mini-PROTEAN® Tetra Cell apparatus (Bio-Rad, Hercules, CA, USA) at 100 V on ice for 1 hour and 15 minutes. Then, the membrane was subjected to Amido Black (Sigma‒Aldrich, St. Louis, Missouri, USA; cat. number: P7170) staining. Then, the membrane was blocked with 5% fat-dry milk in TBS-Tween 20 for 1 hour, followed by overnight incubation with the primary antibody at 4 °C. The following antibodies were used: LC3B 14-16 kDa, 1:1000 dilution (Invitrogen-Thermo Fisher Corporation, Waltham, MA, USA; cat. number: **PA5-32254**); p62/SQSMT 38-47 kDa, 1:1000 dilution (Rockland Immunochimicals, Limerick, PA, USA); and RUNX2 55-62 kDa, 1:1000 dilution (Cell Signaling, Danvers, MA, USA; cat. number 8486). β-Actin (48-55 kDa, 1:10000 dilution; Invitrogen Thermo Fisher Corporation, Waltham, MA, USA; cat. number: MA1-140) served as a housekeeping protein.

## Statistical analysis

The results are presented as the mean ± SD. Statistical analysis was conducted using the Mann–Whitney test for comparisons between two groups or one‐way ANOVA for comparisons involving more than two groups. The analyses were based on experiments conducted six times, and outliers were included in the analyses. SPSS for Windows, version 22.0 (SPSS Inc.), was utilized for the data analysis.
