# Supplementary material for: Unraveling RUNX2 mutation in a cleidocranial dysplasia patient: Molecular insights into osteogenesis and proteostasis
Source: Genes Dis. 2024 Nov 6;12(4):101449. doi: 10.1016/j.gendis.2024.101449 (PMC11960636; doi:10.1016/j.gendis.2024.101449)
Supplement: Multimedia component 2 [file mmc2.docx]

Supplementary Figure Legends

**Figure 1S**: **Experimental workflow of the present study**. One female CCD patient carrying the *RUNX2* c.505C>T mutation and two age-matched healthy female participants were enrolled. Blood samples were collected from the three individuals from which circulating progenitors were selected for osteogenic array analysis. Additionally, iPSC-derived induced mesenchymal stem cells (iMSCs) were obtained from a CCD patient and a healthy control via a Sendai Reprogramming Kit. iMSC colonies expressing the TRA1-60 pluripotency marker were selected after immunocytochemical labeling. iMSCs from CCD patients and healthy controls were further used to study the protein levels of RUNX2, as well as associated gene expression alterations and osteogenic differentiation. In the chest X-ray, the separation of the bone fragments at the mid-third of the right clavicle is indicated with a white arrowhead. Abbreviations: RUNX2, Runt-related transcription factor 2; PBMCs, peripheral blood mononuclear cells; iPSCs, induced pluripotent stem cells; ICC, immunocytochemistry

**Figure 2S: Cleidocranial dysplasia (CCD) patient.** A) X-ray of the lateral projection of the spine; B) X-ray of the anteroposterior projection of the spine; C) X-ray of the lateral projection of the lumbar spine; and D) X-ray of the anteroposterior projection of the chest, where the separation of the bone fragments at the middle third of the right clavicle is indicated with an arrowhead. E) Results of densitometric X-ray absorptiometry (DEXA). (F) Forward genomic DNA sequences of the newly diagnosed *RUNX2* c.505C>T mutation in the patient and its corresponding wild-type sequence. (**G**) The *RUNX2*-coding reference sequence (NM_001024630.3) showing the position of the c.505C>T mutation. (H) Schematic representation of the *RUNX2* gene structure, where the corresponding protein domains and the location of the R169W mutation in the RUNT domain are indicated. Abbreviations: QA, polyglutamine and polyalanine domain; RHD, RUNT homologous domain; NLS, nuclear localization signal; PST, proline/serine/threonine rich region; NMTS, nuclear matrix targeting signal. (Adapted from Jaruga et al. (31)**)**

**Figure S3: Gene expression alterations in a CCD patient carrying a *RUNX2* c.505C>T mutation**. (A) The dendrogram obtained through hierarchical clustering depicts similarities among gene expression patterns. Genes were grouped into clusters according to similarities in their expression patterns, which were computed using Euclidean distances with complete linkage clustering, which considers all pairwise distances between genes. This method highlights any subgroup of genes with similar expression trends. In panels A and B, the TGF-β1-encoding gene is highlighted in red. (B) Quantification of the mRNA levels of TGF-β1 and TGF-βR1 in circulating progenitor cells from a *RUNX2*-mutated CCD patient and healthy donors (*: p < 0.05; **: p < 0.005).

**Figure 4S: Effects of miR9 silencing on bone marrow mesenchymal stem cells. (A)** Western blot analysis of RUNX2 levels after miR-9 silencing. (**B**) RT‒qPCR analysis of the mRNA levels of RUNX2, TGF-β1, and TGF-βR1 after miR9 silencing. (**: *p*< 0.005; ***: *p*< 0.001)

**Figure 5S: Osteogenic differentiation and RUNX2 degradation in CCD cells**. (A) RT‒qPCR analysis of the mRNA levels of the SP7 transcription factor collagen type I/type II alpha (COL1A1/COL2A1), secreted proteins acidic and rich in cysteine (SPARC), and secreted phosphoprotein 1 (SPP1) after 3 and 7 days of differentiation stimulation.

**Figure 6S: Autophagy-related gene levels** RT‒qPCR analysis of the mRNA levels of autophagy-related gene 5 (ATG5) and autophagy-related gene 7 (ATG7) in iMSCs (H) and circulating progenitors.

**Figure 7S. Prediction of the RUNX2 protein interaction network by the STRING portal**. Abbreviations: MAP1LC3B, microtubule-associated protein 1 light chain 3 beta; SQSTM1, sequestosome 1. The protein‒protein interaction (PPI) enrichment p value was 0.00977.
