# Supplementary material for: Unraveling RUNX2 mutation in a cleidocranial dysplasia patient: Molecular insights into osteogenesis and proteostasis
Source: Genes Dis. 2024 Nov 6;12(4):101449. doi: 10.1016/j.gendis.2024.101449 (PMC11960636; doi:10.1016/j.gendis.2024.101449)
Supplement: Multimedia component 3 [file mmc3.pdf]

Genes	controls	CCD
HPRT1	1	1
GUSB	2.785206	3.267627
AHSG	0.000205	0.000304
ALPL	0.001147	0.002945
AMBN	0.00014	0.003273
AMELY	0.00014	0.000304
ARSE	0.003954	0.003114
BGLAP	0.002267	0.003007
BMP1	0.017506	0.032253
BMP2	0.003745	0.004962
BMP3	0.000399	0.000422
BMP4	0.001494	0.000672
BMP5	0.00014	0.000304
BMP6	0.587849	0.262341
BMP7	0.00014	0.000304
BMPR1	0.044083	0.270567
CALCR	0.00105	0.000686
CASR	0.000462	0.000304
CDH11	0.00014	0.016226
COL10A1	0.000489	0.000666
COL11A1	0.00014	0.000304
COL12A1	0.000334	0.001339
COL14A1	0.000328	0.000304
COL15A1	0.00014	0.000413
COL16A1	0.000476	0.001901
COL17A1	0.00014	0.000304
COL18A1	0.015898	0.084844
COL19A1	0.002159	0.002474
COL1A1	0.000123	0.000304
COL1A2	3.4E-05	0.000297
COL2A1	0.00014	0.000304
COL3A1	0.000169	0.000304
COL4A3	0.00174	0.001954
COL4A4	0.000249	0.000464
COL4A5	0.00014	0.000304
COL5A1	0.00022	0.00031
COL7A1	0.002424	0.002496
COL9A2	0.032797	0.03476
COMP	0.00014	0.000304
CSF2	0.001548	0.003084
CSF3	0.00014	0.000276
DMP1	0.00014	0.000304
EGF	0.443759	0.201971
EGFR	0.00014	0.000286
ENAM	0.000255	0.0003
FGF1	0.00014	0.000304
FGF2	0.00807	0.006758
FGF3	0.00014	0.000304
FGFR1	0.105245	0.132159

FGFR2	0.001227	0.003577
FGFR3	0.00014	0.000329
FLT1	0.00282	0.001273
GDF10	0.000284	0.001771
IBSP	0.00014	0.000304
IGF1	0.000133	0.000193
IGF1R	2.215301	4.611516
IGF2	0.000717	0.00263
MGP	0.002947	0.004581
MINPP1	0.445599	0.901429
MMP13	0.00014	0.000304
MMP2	0.001989	0.002547
MMP8	0.00014	0.000304
MSX1	0.00014	0.000304
MSX2	0.000786	0.002236
DSPP	0.000278	0.000304
PDGFA	0.820282	0.460801
PHEX	0.039422	0.0316
RUNX2	1.043166	1.269936
SMAD1	0.274529	0.317527
SMAD2	1.578753	1.864712
SMAD3	0.348262	0.57873
SMAD4	1.3915	3.010452
SMAD5	0.918278	1.744789
SMAD6	0.016708	0.020928
SMAD7	0.204313	0.583074
SMAD9	0.000122	0.001236
SOST	0.00014	0.000304
SOX9	0.00014	0.000304
SèARC	2.086417	2.898737
SPP1	0.002119	0.009541
STATH	0.00014	0.000304
TFIP11	1.494348	2.213088
TGFB1	19.56842	23.19992
TGFB2	0.068732	0.03715
TGFB3	0.010743	0.026231
TGFBR1	1.545682	3.843644
TGFBR2	4.77406	5.395139
TUFT1	0.02584	0.038899
TWIST1	0.000231	0.000435
TWIST2	0.000145	0.000412
VDR	0.991812	0.975484
VEGFA	0.481337	0.502414
VEGFB	0.796152	2.305363
VEGFC	0.054617	0.014501
